# A centromere satellite concomitant with extensive karyotypic diversity across the *Peromyscus* genus defies predictions of molecular drive

**DOI:** 10.1007/s10577-019-09605-1

**Published:** 2019-02-15

**Authors:** Brendan M. Smalec, Thomas N. Heider, Brianna L. Flynn, Rachel J. O’Neill

**Affiliations:** Institute for Systems Genomics and Department of Molecular and Cell Biology, University of Connecticut, 67 North Eagleville Road, Unit 3127, Storrs, CT 06269 USA

**Keywords:** Centromere, Satellite DNA, *Peromyscus*, Chromosome rearrangements, Concerted evolution, Molecular drive

## Abstract

A common feature of eukaryotic centromeres is the presence of large tracts of tandemly arranged repeats, known as satellite DNA. However, these centromeric repeats appear to experience rapid evolution under forces such as molecular drive and centromere drive, seemingly without consequence to the integrity of the centromere. Moreover, blocks of heterochromatin within the karyotype, including the centromere, are hotspots for chromosome rearrangements that may drive speciation events by contributing to reproductive isolation. However, the relationship between the evolution of heterochromatic sequences and the karyotypic dynamics of these regions remains largely unknown. Here, we show that a single conserved satellite DNA sequence in the order Rodentia of the genus *Peromyscus* localizes to recurrent sites of chromosome rearrangements and heterochromatic amplifications. Peromyscine species display several unique features of chromosome evolution compared to other Rodentia, including stable maintenance of a strict chromosome number of 48 among all known species in the absence of any detectable interchromosomal rearrangements. Rather, the diverse karyotypes of Peromyscine species are due to intrachromosomal variation in blocks of repeated DNA content. Despite wide variation in the copy number and location of repeat blocks among different species, we find that a single satellite monomer maintains a conserved sequence and homogenized tandem repeat structure, defying predictions of molecular drive. The conservation of this satellite monomer results in common, abundant, and large blocks of chromatin that are homologous among chromosomes within one species and among diverged species. Thus, such a conserved repeat may have facilitated the retention of polymorphic chromosome variants within individuals and intrachromosomal rearrangements between species—both factors that have previously been hypothesized to contribute towards the extremely wide range of ecological adaptations that this genus exhibits.

## Introduction

In most eukaryotic genomes, large arrays of tandem repeats are often confined to functionally defined subregions within chromosomes, such as the centromere and telomere. Exemplars are the virtually ubiquitous satellite DNA sequences that are present in tandem arrays in the centromeres of many eukaryotes. While maintaining a functional centromere is necessary to sustain chromosome stability by mediating proper kinetochore and microtubule attachment, the satellite DNA sequences that underlie this region are surprisingly among the most rapidly evolving portions of the eukaryotic genome (Henikoff et al. 2001), largely affected by processes such as molecular drive (Dover 1982) and centromere drive (Henikoff and Malik 2002; Malik and Henikoff 2002).

Satellites and other centromeric repeats in the mammalian genome evolve via concerted evolution, a non-independent process of molecular drive by which a species or population maintains an unusually high intraspecific repeat homogeneity and, concomitantly, a high interspecific heterogeneity (Dover 1982). Several mechanisms, including nonhomologous and/or unequal crossing over (Smith 1976), replication slippage (Walsh 1987), gene conversion (Shi et al. 2010), and rolling circle amplification and subsequent reinsertion (Bertelsen et al. 1982; reviewed in Gaubatz 1990), allow specific satellite DNA sequences to quickly spread throughout the genome and replace other repetitive sequences, despite their inherent inability to directly transpose themselves. Centromere drive may also affect the rapid satellite divergence between species as it is hypothesized that satellites and their DNA binding partners at the centromere, such as the centromere-specific histone centromere protein A (CENP-A), are in conflict with one another that manifests during female meiosis (Malik and Henikoff 2002). Further challenging the presumption that the centromere would be selected to remain a static region of the genome in order to support genome stability is the finding that it often is a hotspot for chromosome rearrangements, including translocations, inversions, and fusions (reviewed in Brown et al. 2012). Thus, the centromere is thus a host to both rapid nucleotide and karyotypic variation.

The “library hypothesis” (Salser et al. 1976) provides another explanation for how satellite DNA content at the centromere may diverge rapidly among closely related species. According to this hypothesis, extant but distinct centromeric repeats—which comprise the satellite “library”—may independently expand or contract in copy number in different evolutionary lineages. These alterations in the relative proportions of sequences at the centromere may result in production of divergent or unique centromere profiles for different species, and even different chromosomes within species. For example, human and chimpanzee alpha satellite HORs were found to share ~ 75% sequence identity and phylogenetic analyses of primate alpha satellites indicated each primate species examined carries evolutionarily distinct satellites (Alkan et al. 2007). Several studies support this hypothesis across widespread eukaryotic taxa, including the discovery of four distinct centromere satellites in four species of *Palorus* (beetle) (Mestrovic et al. 1998). More recently, species-specific satellite DNA amplification has also been observed in *Leporinus elongatus* (tropical freshwater fish) (da Silva et al. 2013), *Aotus azarae* (Azara’s owl monkey) (Prakhongcheep et al. 2013), and *Oryza brachyantha* (wild African rice) (Lee et al. 2005). Moreover, divergent satellite sequences also define the centromeres of specific chromosomes in *Cercopithecus pogonias* and *Cercopithecus solatus* (Old World monkeys) (Cacheux et al. 2018), *Bactrocera oleae* (olive fruit fly) (Tsoumani et al. 2013), *Cricetulus griseus* (Chinese hamster) (Faravelli et al. 1998), and *Solanum tuberosum* (potato) (Gong et al. 2012). Hence, rapid satellite DNA divergence is a common feature across eukaryotic lineages and swift changes in centromere DNA content can contribute to reproductive isolation that may drive speciation (Ferree and Barbash 2009).

Following the premise of the library hypothesis, it would reason that chromosome rearrangements affecting centromeric heterochromatin would cause a divergence in centromere satellite content as these events create an opportunity for divergent, or less abundant, repeats present in the library to become amplified within the rearranged chromosome and, through the mechanisms of molecular drive, the entire genome. Thus, this model predicts that alterations in the relative copy numbers of extant, variant satellite sequences should accompany karyotypic evolution. In support of this hypothesis, it has been found that dramatic expansions and contractions of three separate satellites in the *Macropus* genus (wallabies) are concurrent with specific chromosome rearrangements, notably translocations and fusions—both involving the centromere (Bulazel et al. 2007). A similar phenomenon has been reported in the family Bovidae, which includes cattle, goat, and sheep. Karyotypic evolution of these genera primarily progresses via fusions of two acrocentric chromosomes at the centromere, which is accompanied by a change in a relative copy of the centromeric satellite sequences on the resulting biarmed chromosome (Chaves et al. 2003; Chaves et al. 2000; D’Aiuto et al. 1997). These data support hypothesis that chromosome rearrangements, while not exclusively necessary for the rapid evolution of centromeric DNA, may serve as a means by which centromere satellite evolution is facilitated.

One genus that is particularly useful for studying dynamic centromere and heterochromatin restructuring, chromosome evolution, and rapid speciation in parallel is *Peromyscus*. This genus encompasses at least 56 species and serves as a valuable model system for a broad set of applications, including studies in behavior, ecology, evolution, and disease systems (reviewed in Bedford and Hoekstra 2015; Shorter et al. 2012). All species share a 2*n* = 48 karyotype (Arakaki and Sparkes 1967; Cross 1938), although both interspecific and intraspecific variations among chromosomes are typified by pericentric inversions and heterochromatic additions at common, known sites of rearrangement (Duffey 1972; Pathak et al. 1973). As a result, the fundamental number (number of chromosome arms) ranges from 56 to 96 across the genus (Pathak et al. 1973). Thus, while large-scale chromosome rearrangements do not distinguish diverse karyotypes within this genus, changes in heterochromatin content and pericentromeric arrangement characterize chromosome divergence across *Peromyscus* species.

Here, we present both cytogenetic and sequence analyses of satellite DNA within seven species of *Peromyscus* representing each of the major clades within the genus (Bradley et al. 2007). Previous work by Louzada et al. (2015) led to the identification of a *Peromyscus* satellite sequence, *Peromyscus maniculatus* satellite repeat (PMsat), that is present in high copy number in *Peromyscus eremicus*. Moreover, this study employed a computational approach to show that PMsat is found in tandem arrays in *Peromyscus maniculatus*, likely representing a centromeric repeat in this species (McNulty and Sullivan 2018; Plohl et al. 2012). Extending this earlier work, we set out to test whether molecular drive characterizes satellite repeats across a species complex that is exemplified by large-scale changes in the copy number and distribution of satellite-rich heterochromatin. We find that a single, defined centromeric satellite is a key component of heterochromatin additions and rearrangements across the *Peromyscus* genus and that this satellite displays a remarkable level of evolutionary conservation, contrary to predictions of the various forms of drive that may act on centromeric DNAs. We propose that the conservation of this element may contribute to the nearly unparalleled level of intraspecific and interspecific karyotypic variations exhibited in the *Peromyscus* genus. While the satellite DNA itself may not be actively participating in these chromosome rearrangements, the conservation of homogenous tandem arrays may facilitate the evolution of diverse karyotypes.

## Methods

### Cell culture, cytogenetics, and genomic DNA extractions

One ear punch for an individual from the following species was obtained from the *Peromyscus* Genetic Stock Center at the University of South Carolina: *Peromyscus leucopus* (LL), *Peromyscus aztecus hylocetes* (AM), *Peromyscus melanophrys xenerus* (XZ), *Peromyscus californicus insignis* (IS), *P. eremicus* (EP), *P. maniculatus bairdii* (BW), *P. maniculatus sonoriensis* (SM2), *Peromyscus polionotus subgriseus* (PO), and a BW × PO hybrid individual. Ear fibroblast cell lines were established using standard collagenase methods. An ear fibroblast cell line from *Cricetulus griseus* (Chinese hamster) was utilized as a closely related outgroup species as in Mlynarski et al. (2010). Metaphase chromosomes were prepared from primary fibroblasts as per Brown et al. (2002), and slides were dropped as per standard protocols. High molecular weight genomic DNA was obtained by phenol/chloroform extraction and ethanol precipitation using standard protocols from cell pellets for all species, with the exception of *Peromyscus leucocephalus-subgriseus* (LP), from which DNA was isolated from liver tissue.

### PCR amplification

To determine if the satellite DNA sequence first identified by Louzada et al. (2015) in *P. eremicus* was also present in the genome of *P. leucopus*, the following PCR primers were designed with Primer3 (Untergasser et al. 2012): 5′-ACAGGAGCTTCTCTTCAGTCC-3′ and 5′-AAGCAGAGTGTTTTGGGTGT-3′. PCRs were performed using gDNA from *P. leucopus*, products were subcloned using the StrataClone PCR cloning kit (Agilent) as per the manufacturer’s protocol, and recombinant colonies were sequenced using BigDye^®^ Terminator v3.1 Cycle Sequencing Kit (Thermo Fisher) on an Agilent 3130 Genetic Analyzer.

### Fluorescence in situ hybridization

Slides were pretreated with 0.1 mg/ml RNase A and 2× saline sodium citrate (SSC) at 37 °C for 15 min and rinsed four times for 2 min in 2× SSC, followed by a protease treatment as follows: CytoZyme Stabilized Pepsin for 10 min at 37 °C (for *P. aztecus hylocetes* species hybridization) or 0.2 N HCl for 20 min at room temperature (all other species). The slides were then dehydrated, air-dried, and denatured for 2 min in 70% formamide and 2× SSC at 72 °C before hybridization with the denatured probe.

Target DNA was amplified and labeled with biotin-16-dUTP (Roche) via PCR from *P. leucopus* genomic DNA using the aforementioned primers. The labeled probe from *P. leucopus* was used in fluorescence in situ hybridization (FISH) for all species with the following exceptions. Probes were prepared from *P. aztecus hylocetes*, *P. melanophrys xenerus*, and *C. griseus* genomic DNA using the same primers as above and were hybridized conspecifically to metaphase chromosomes as hybridization signal was not detected reliably across different metaphase spreads of these species using the *P. leucopus* probe. In all cases, hybridization probes were prepared by precipitating 500 ng of labeled DNA and 25 μg salmon sperm DNA and resuspending the DNA in Hybrisol VII (MP Biomedicals). Probes were denatured at 80 °C for 10 min and subsequently hybridized to the denatured slides for about 18 h at 37 °C.

Post-hybridization washes were performed for 5 min in 2× SSC at 45 °C followed by washing two times for 5 min in 1× SSC at 72 °C for all species, except *P. aztecus hylocetes* (0.5× SSC at 72 °C) due to nonspecific background. Slides were blocked in 4× SSC, 0.2% Tween 20, and 5% bovine serum albumin for 30 min at 37 °C, and probes were detected using a 1:400 dilution of avidin Texas Red (Invitrogen) in 4× SSC, 0.2% Tween 20, and 5% bovine serum albumin for 20 min. Excess detection reagents were removed by washing three times for 5 min in 4× SSC and 0.2% Tween 20 at 45 °C. Metaphases were counterstained with a dilution of 1:4 DAPI:Vectashield (Vector Laboratories). Images were captured using an Olympus AX-70 microscope equipped with Genus imaging software (Applied Imaging). A minimum of 20 cells was assessed for each species. Inverted DAPI images were used to identify chromosome pairs within each karyotype.

### Southern blotting

Approximately 10 μg of high molecular weight DNA was digested overnight at 37 °C in separate reactions for each species with *Msp*I (New England Biolabs). Restriction digests were electrophoresed on a 0.8% agarose gel overnight at 30 V and transferred to a Hybond N^+^ nylon membrane (Amersham) by standard methods. The satellite probe was amplified as above using *P. leucopus* genomic DNA, the product was labeled with ^32^P-dCTP using random primers and hybridized to the membrane in Church’s solution (500 mM Na_2_HPO_4_, 7% SDS, 1 mM EDTA) at 60 °C overnight. Post hybridization, the membrane was washed in 2× SSC and 0.1% SDS at room temperature for 10 min, 2× SSC and 0.1% SDS at 60 °C for 15 min, and 1× SSC and 0.1% SDS 60 °C for 15 min. The membrane was exposed to X-ray film for 18 h at − 80 °C and then developed.

### Sequencing and data analysis

The satellite repeat monomer was amplified via PCR in all species with primers designed to obtain the following complete monomer sequence: 5′-CGCGTCTGTTCCCAGCAA-3′ and 5′-AGTGCTATTTGCACTGTKTAT-3′. The PCR reaction was run on an agarose gel, and DNA from the second largest band (~ 680 bp), representing multiple monomers, was extracted using the QIAquick Gel Extraction Kit (Qiagen) as per the manufacturer’s protocol. The PCR products were subcloned using the StrataClone PCR cloning kit (Agilent) as per the manufacturer’s protocol, and recombinant colonies were sequenced using BigDye^®^ Terminator v3.1 Cycle Sequencing Kit (Thermo Fisher) on an ABI 3130 Genetic Analyzer. At least 10 clones were sequenced per species; sequences were trimmed of vector and primer sequences and a single monomer within the primers used for PCR was selected using Geneious 8.1 (Biomatters) and aligned using MUSCLE (Edgar 2004) and ClustalW (Larkin et al. 2007) implemented in Geneious 8.1. Sequences were searched for functional motifs using the MEME Suite (Bailey et al. 2009). All satellite monomer clones are reported in GenBank under accession numbers KX555281–KX555350.

### Acquisition and filtering of next-generation sequencing libraries

Low-coverage next-generation sequencing (NGS) data available for four species of *Peromyscus* (*P. maniculatus* SRR1012309, *P. polionotus* SRR545676, *P. californicus* SRR545682, and *P. leucopus* SRR545963) was downloaded using fastq-dump (with parameters “--split-3 --gzip -W --read-filter pass”) and the paired reads were merged using pandaseq (default parameters). The files were then masked using RepeatMasker and the previously identified PMsat sequences (with parameters “-nolow -no_is -norna -gff -e ncbi -lib clones.fasta”). Only reads that contained 99% repetitive sequences were retained in an effort to focus on tandemly arrayed sequences rather than solo, and likely more divergent and noncentromeric monomers. The remaining reads were then mapped using BWA mem (default parameters) against the clone library derived from Sanger sequencing, and a 25-bp sliding window was used to find the region where the highest number of merged reads start mapping.

### Clustering and quantification of NGS reads: repeatConnector

We developed a tool, repeatConnector, to mine existing next-generation sequencing data in the SRA database to generate a minimum spanning tree of clusters of repetitive sequences. The tool is designed to compare similar repeats across different datasets, in this case different species. To accomplish this, it first merges paired end reads using pandaSeq (Masella et al. 2012) and then filters the merged reads using a user-supplied fasta file containing the repeat of interest. The filtering is done to reduce the computational time required to cluster the merged reads. Using BWA (Li 2013) and RepeatMasker, the script removes any read that has no similarity to the user-supplied repeat library and then uses cd-hit-est (Fu et al. 2012) for clustering the reads that passed the filtering steps clustered at each of the following rates: 95%, 97%, and 99% similarity (with parameters “-M 50000 -s 0.8 -d -g 1”). The similarity between all clusters was calculated using a matcher from the EMBOSS suite (Rice et al. 2000). To calculate the composition of every cluster, the best hit from BWA to the initial fasta file was used to identify the most similar source sequence. The raw counts, counts normalized to the initial number of reads, and counts normalized to the number of reads passing the quality filters were also calculated. Shannon’s *H* was calculated for each cluster and for each method of counting merged reads. The above information about each cluster was then used to generate and display the minimum spanning tree in R using the igraph package (Csardi and Nepusz 2006). Scripts and background information on the *repeatConnector* can be found at https://gitlab.com/roneill_lab/repeatConnector.

## Results

### Identification of a conserved heterochromatic satellite repeat

**Fig. 1:**
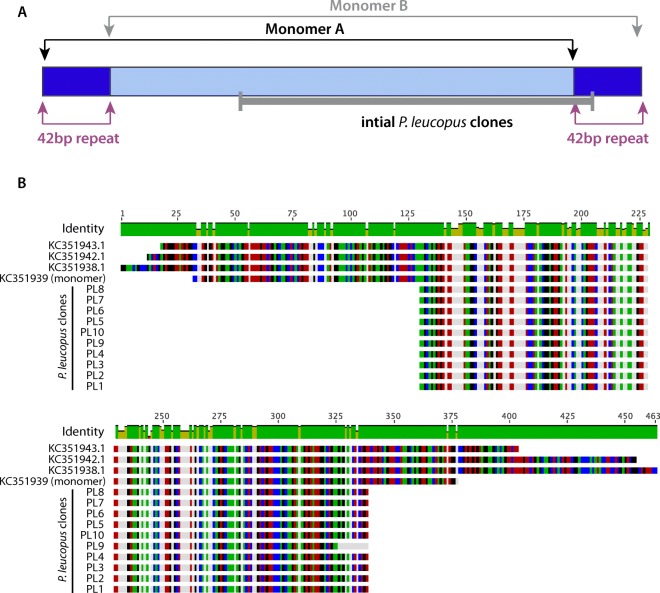
*P. leucopus* shares the PMsat satellite with *P. eremicus*. **a** Schematic of the relationship between monomer A of PMsat identified from *P. eremicus* relative to monomer B (targeted herein) and the shorter sequence initially identified in *P. leucopus*. **b** Sequence identity of the *P. leucopus* clones compared to partial and complete monomers previously identified from *P. eremicus* (accession numbers KC351943.1, KC351942.1, KC351938.1, and KC351939) (Louzada et al. (2015). Similarities among sequences are coded by colors as follows: A is green, C is blue, G is black, T is red, and white is a disagreement among the sequences

A sequence sharing identity to PMsat, a satellite previously identified in *P. eremicus* (Louzada et al. 2015), was isolated by PCR in *P. leucopus* and confirmed via Sanger sequencing (Fig. 1). A total of 10 clones aligned to the previously annotated PMsat from *P. eremicus* (accession numbers KC351943.1, KC351942.1, and KC351938.1) and a single monomer A (KC351939) with an overall pairwise identity of 94.9% to a region slightly smaller than a full monomer (Fig. 1a, b). Given our finding that this *P. eremicus* satellite is conserved in *P. leucopus* and previous work demonstrating PMsat is found in divergent species of Rodentia (*Cricetus cricetus*) (Louzada et al. 2015), we set out to test whether the sequence was a primary component of heterochromatin blocks and/or centromeric regions in other *Peromyscus* species via fluorescence microscopy using the complete monomer (monomer B).

**Fig. 2:**
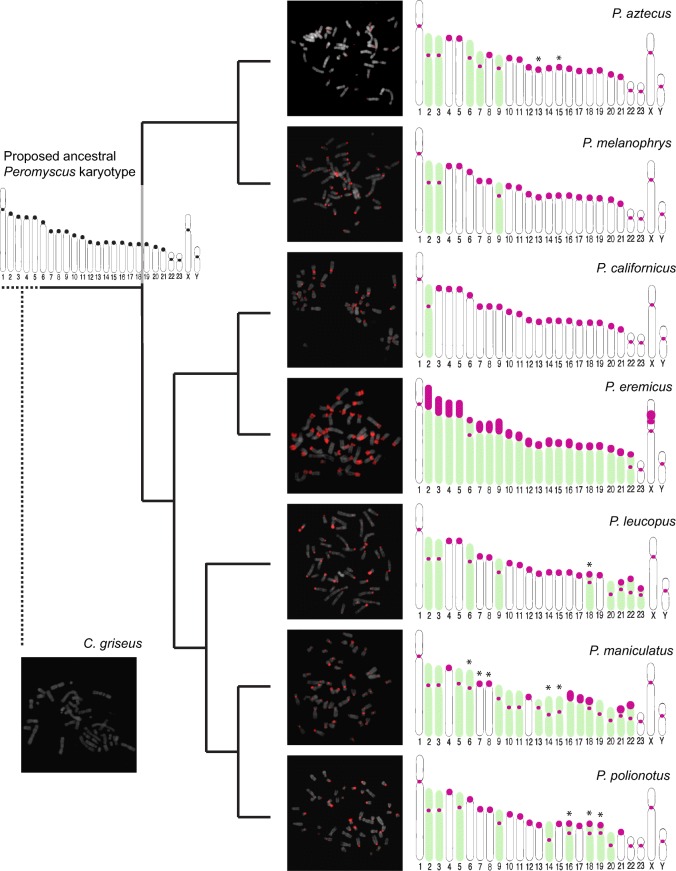
Satellite probe hybridizes to centromeres of all species of *Peromyscus* but varies at telomeric locations. A fluorescent probe was prepared and hybridized to metaphase spreads, and the genomic distribution of the satellite on each chromosome was recorded in each species. A representative FISH image is shown for each species (DAPI-stained chromosomes are gray, and probe signal is red), and the results are summarized in the ideogram. No hybridization was observed in *C. griseus*. Green-shaded chromosomes represent those that have diverged from the ancestral karyotype (shown at the base of the tree, centromeres are denoted by black-filled areas), indicating that it has experienced interspecific rearrangement. Asterisks denote chromosomes known to exist in polymorphic forms within an individual of the respective species. Phylogenetic relationships among species shown to the left and based on Bradley et al. (2007)

The satellite sequence hybridized to the centromeres of all chromosomes of *P. leucopus*, in addition to the axial telomeres (i.e., the telomere of the short arm of a chromosome) of some of the biarmed chromosomes (Fig. 2). To qualitatively compare the conservation of PMsat across the *Peromyscus* genus, its chromosomal location was visualized through FISH using cells from seven additional species as well as an outgroup species, the Chinese hamster (*C. griseus*). Across all *Peromyscus* samples, the PMsat sequence was found at the centromeres of all chromosomes, although the variation among species was observed in both copy number and presence at noncentromeric heterochromatic regions, such as the telomere (Fig. 2).

For each species, the following positive hybridization signal was observed: in *P. leucopus*, centromeric signal across all chromosomes with four chromosomes showing signal at both the centromere and the axial telomere (chr. 18, 21–23); in *P. aztecus*, *P. melanophrys*, and *P. californicus*, all chromosomes with signal restricted to the centromere; and in *P. eremicus*, 24 biarmed chromosome pairs, two autosomal pairs with signal restricted to the centromere (chr. 1, 23), two autosomal pairs with signal at both the centromere and the axial telomere (chr. 6, 22), and 19 autosomal pairs with signal extending from the centromere to the axial telomere (chr. 2–5, 7–21). The X chromosome displayed signal both at the centromere and at two other blocks along a single arm, and the Y chromosome exhibited signal at the centromere only. In *P. maniculatus*, all chromosomes carry signal at the centromere, three pairs with signal at both the centromere and the axial telomere (chr. 18, 21, 22), and two pairs with signal extending from the centromere to the axial telomere (chr. 16, 17). In *P. polionotus*, all chromosomes carry signal at the centromere and three with signal at both the centromere and the axial telomere (chr. 16, 18, 19). In *C. griseus*, no specific hybridization to chromosomes was observed.

### Repeat organization in the *Peromyscus* genome

**Fig. 3:**
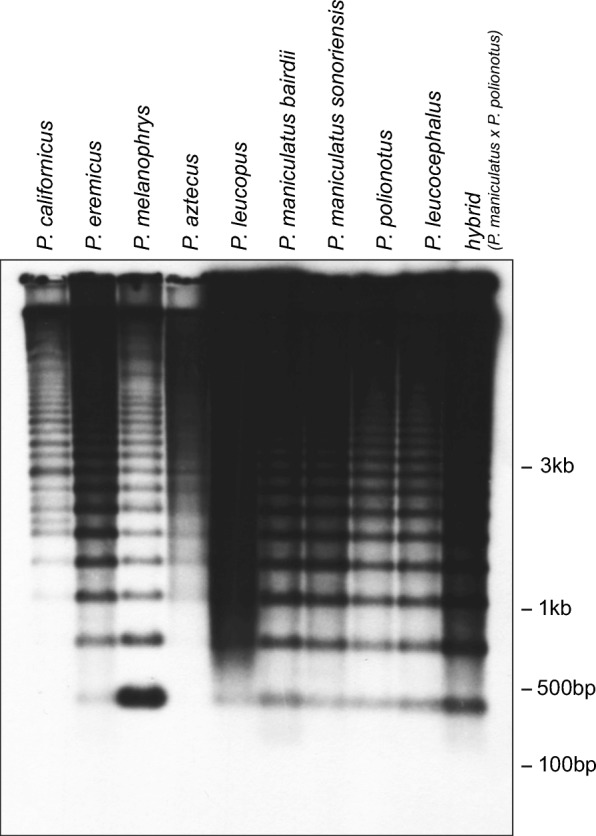
Southern analysis confirms tandem arrangement of satellite in all species of *Peromyscus*. Genomic DNA was digested with *Msp*I, electrophoresed, transferred to a membrane, and probed with the satellite sequence. A ladder-like hybridization pattern observed in all species confirms the tandem arrangement of the satellite. Lanes are as follows from the left: *P. californicus*, *P. eremicus*, *P. melanophrys*, *P. aztecus*, *P. leucopus*, *P. maniculatus bairdii*, *P. maniculatus sonoriensis*, *P. polionotus subgriseus*, *P. leucocephalus-subgriseus*, and *P. maniculatus* × *P. polionotus* hybrid. Marker sizes are shown to the right

To characterize the length and arrangement of this heterochromatic repeat, Southern blotting was performed across the species that showed high copy numbers of PMsat in defined heterochromatic blocks in the karyotype, thus excluding the outgroup *C. griseus*, which lacked hybridization signal via FISH. Two additional samples were included in this analysis: LP and a *P. maniculatus* × *P. polionotus* hybrid. A ladder-like hybridization pattern was observed across all individuals with a conserved periodicity and monomer size of about 350 bp (Fig. 3), banding patterns that are consistent with a satellite DNA sequence present in a homogenized tandem array (reviewed in Garrido-Ramos 2017). This data suggests that the PMsat satellite is a conserved tandem repeat across all *Peromyscus* species assayed, with remarkable conservation of restriction site positions and periodicity of the satellite monomer.

**Fig. 4:**
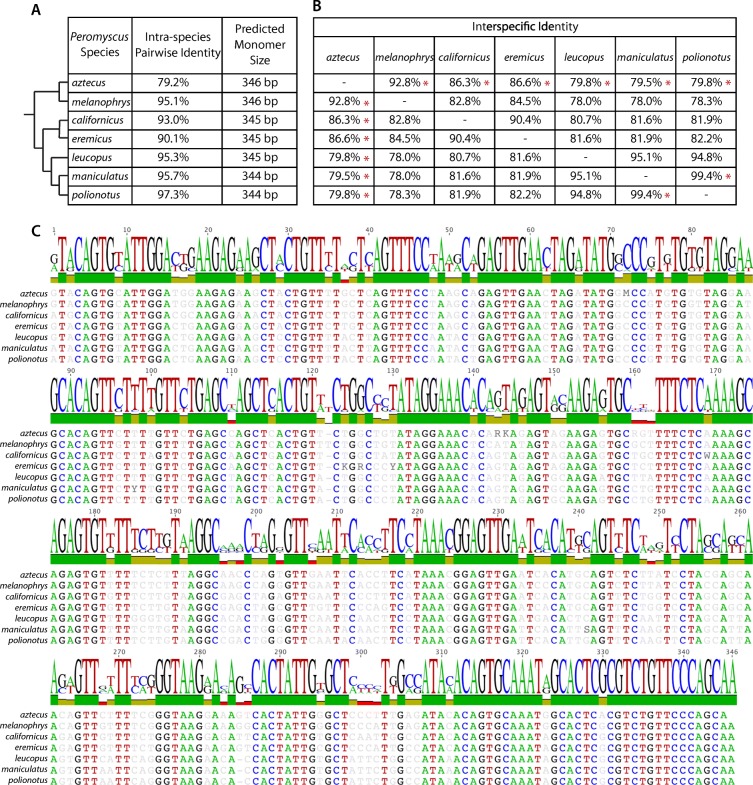
Sequence of satellite monomer does not exhibit species-specific patterns of nucleotide identity. **a** The intraspecific identity of the satellite and the predicted monomer length were calculated by aligning multiple clones within each respective species. **b** The interspecific identity of the satellite was calculated by aligning the consensus sequences generated from each species. Asterisks indicate instances where interspecific homology is greater than intraspecific homology, suggesting that the influence of concerted evolution is limited. **c** Alignment of monomer consensus sequences from each species shows identity throughout the entire satellite, with no specific region of the sequence displaying higher or lower levels of sequence conservation

To quantitatively characterize the nucleotide identity across *Peromyscus* species and test for evidence of molecular drive, the intraspecific identity of the satellite was compared to interspecific identity in two different ways: first, a set of PCR amplicons for each species was used to determine inter- vs intraspecific identity. This approach is based on the assumption that the most predominant satellite variant within a species is more likely to reach exponential amplification within a single PCR, thus biasing these data towards assessing sequences with lower levels of intraspecific variation. Indeed, in most cases, the intraspecific sequence identity was greater than the interspecific identity of closely related species, indicating that concerted evolution of the satellite may be taking place, at least on common variants (Fig. 4). The monomer size and pairwise identity within each species were as follows: in *P. aztecus*, 79.2% (346 bp); in *P. melanophrys*, 95.1% (346 bp); in *P. californicus*, 93.0% (345 bp); in *P. eremicus*, 90.1% (345 bp); in *P. leucopus*, 95.3% (345 bp); in *P. maniculatus*, 95.7% (344 bp); and in *P. polionotus*, 97.3% (344 bp) (Fig. 4a). All species-specific consensus sequences share between 80% (*P. leucopus*) and 96% (*P. eremicus*) identity to three clones sequenced in *P. eremicus* by Louzada et al. (2015) (accession numbers KC351938, KC351942, and KC351943). To estimate the interspecific degree of conservation of the satellite to facilitate comparisons to known phylogenetic relationships among species, each intraspecific sequence was subject to multiple pairwise alignments to each PMsat sequence for every species (Fig. 4b, c).

Several species showed notable exceptions to predictions that interspecific differences would be greater than intraspecific differences. Copies of *P. aztecus* PMsat showed much lower intraspecific satellite identity than observed in either intra- or interspecific comparisons among all other species. Additionally, the interspecific identity of the satellite between *P. maniculatus* and *P. polionotus* is higher than the intraspecific identity of either of the species, perhaps reflective of their recent divergence (estimated at 1.5–3 million years ago) (Platt et al. 2015).

Previously developed bioinformatic tools afford the opportunity to mine next-generation sequencing data for repeats and the relationship of repeat variants within a species (e.g., Repeat Explorer) (Novak et al. 2013) or to detect satellites and derive estimates for intragenomic variation (e.g., satMiner) (Ruiz-Ruano et al. 2016). However, we required a tool that would facilitate the screening of next-generation sequencing datasets for specific satellite sequences and the subsequent analyses of phylogenetic relationships among these satellite repertoires across different species. To further examine the relationship of sequence identity of the PMsat satellite within and between species, we employed our newly developed tool, *repeatConnector*, using available next-generation sequencing datasets for species included in our study (*P. maniculatus*, *P. polionotus*, *P. californicus*, and *P. leucopus*) and representing all but one clade within the genus (no data were available for the *P. melanophrys-aztecus* clade). Paired end, whole-genome shotgun Illumina reads were trimmed for quality, merged, and masked against a de novo repeat database including the PMsat sequences obtained from all species via PCR. Reads with a minimum of 99% repeat content were retained to narrow our focus to those repeats present in tandem arrays and thus more likely to be within the CENP-A-delimited region of the centromere rather than pericentric and degenerated variants (reviewed in Brown and O’Neill 2014; Garrido-Ramos 2017; Plohl et al. 2012). Surviving reads were clustered using cd-hit-est at 95%, 97%, and 99% similarity. To generate the similarity score between each cluster, a matcher was used to calculate the percent similarity among the sequences for each cluster. The sequence identity of satellites within each cluster was calculated using the best hit from BWA alignments among libraries to identify the most similar clone and thus define cluster relationships.

**Fig. 5:**
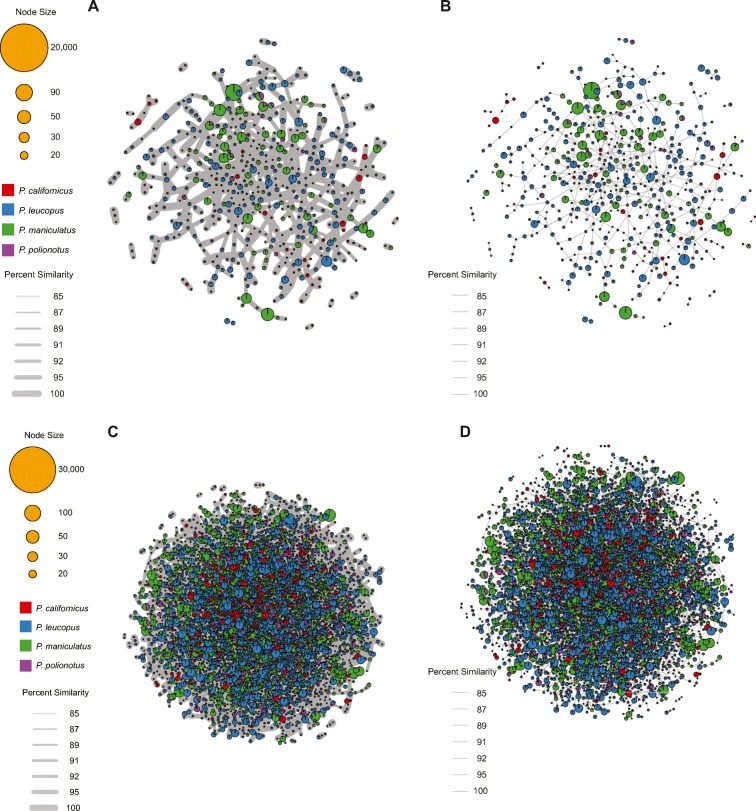
Satellite cluster analyses across *Peromyscus* lack structure. RepeatConnector cluster analysis of satellites isolated from *P. maniculatus*, *P. polionotus*, *P. californicus*, and *P. leucopus* shotgun sequencing data. Reads containing at least 99% satellite sequence identity to centromeric satellites were clustered by sequence identity across all reads. Clusters (shown as nodes) representing 97% identity (**a**, **b**) and 95% identity (**c**, **d**) were annotated based on the originating sequencing library (colors). Clusters containing reads from more than one species-specific library are represented by pie graphs, with the colors therein denoting the species library. The size of each node indicates the number of sequences therein. The sequence identity among clusters is indicated by line thickness, encompassing a range of identities from 85 to 100%, with most falling between 95 and 100%. **a**, **c** The clusters with connections represented by lines whose thickness is represented by the percent divergence. **b**, **d** The same clusters with connections represented by lines whose thickness is proportionally reduced for visual clarity

Models of molecular drive predict that satellite variants, represented by nodes, within each species would cluster together and separately from clusters of satellites from other species. However, we find that this is not the case, at least for the clades assessed; clusters from different species representing different clades within *Peromyscus* are intermingled, as seen by different colored circles within the same network, show high sequence identities between nodes of different colors, most often between 95 and 100% identity, and clear species-specific clusters were not identifiable. Moreover, in many cases, cluster nodes were comprised of satellites from different species (pie graphs at nodes), indicating that interspecific variation is lower than intraspecific variation. Across all clades assessed, we find this pattern to hold true when using low similarity thresholds (97% and 95%, Fig. 5a–d), which would pick up more divergent satellites as well as variants that are more likely to have signatures of concerted evolution within any given dataset. Thus, the ancestor to the *P. maniculatus-polionotus*, *P. leucopus*, and *P. californicus-eremicus* clades, representing all but one of the major clades within *Peromyscus*, likely carried a limited (i.e., low diversity) satellite library. While the clone-based data for the *P. melanophrys*/*aztecus* clade indicates a similar pattern of limited diversity would be expected from deep sequencing of satellite libraries for species within this clade, we lack next-generation sequencing data for these species and thus can only infer that the low diversity of satellites across all clades is an ancestral characteristic.

**Fig. 6:**
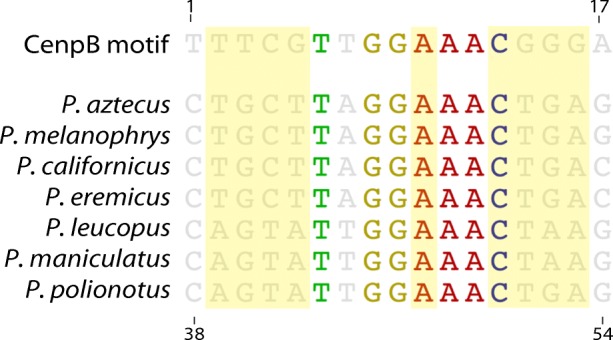
Divergent CENP-B DNA-binding motifs are present within *Peromyscus* satellite monomers. Alignment of the 17-bp consensus CENP-B DNA binding motif and consensus satellite monomers across *Peromyscus*. The nine bases required for CENP-B binding in human are shaded in yellow; sites conserved among all sequences are shown in bold

Given the conservation of this satellite as a tandemly arrayed monomer and a component of the centromere region of each chromosome across all species examined, we tested whether this satellite carries detectable signals of centromere protein binding activity, demarcating PMsat as a component of chromatin involved in kinetochore assembly. Using FIMO (Bailey et al. 2009), we searched for evidence of a centromere protein B (CENP-B) binding box, a feature that is prevalent in many functional centromeric satellites, including the human centromeric alpha satellite (Masumoto et al. 1989). Despite the conservation of this satellite in large arrays at centromeres, we did not detect the canonical CENP-B motif. The strongest alignment of the CENP-B motif (*p* = 0.00133, *q* = 0.686) was found in the same location of each satellite but yielded an alignment in which only six of the nine bases that are critical for pairing (reviewed in Masumoto et al. 2004) are conserved in four species (*P. aztecus*, *P. melanophrys*, *P. californicus*, *P. eremicus*), only five of the nine conserved in *P. polionotus*, and four of the nine conserved in *P. leucopus* and *P. maniculatus* (Fig. 6). Based on the consensus CENP-B binding activity known for human and mouse, this motif is predicted to be nonfunctional. However, *Peromyscus*-specific binding activity for CENP-B is currently unknown as CENP-B antibodies for this species are not available; it is possible that a diverged consensus sequence may be required for functional activity in this lineage.

## Discussion

Chromosome rearrangements involving centromeric or telomeric heterochromatin are predicted to lead to divergence in satellite monomer sequence as these DNA breaks can create an opportunity for mutated or less abundant repeats to become amplified in the new chromosome and, eventually—through the mechanisms of concerted evolution—the entire genome. Given the conservation of this satellite sequence across all major clades within the *Peromyscus* genus, the predominant satellite at *Peromyscus* centromeres does not follow the predictions of the “library model” of satellite DNA evolution (Salser et al. 1976)—a hypothesis that would explain high centromere sequence divergence between closely related but karyotypically distinct species, such as that observed in the Peromyscine species. Rather than different repetitive sequences being selectively amplified in various *Peromyscus* genomes through the process of molecular drive (Dover 1982), the same sequence is found at the centromeres of all *Peromyscus* species studied with little variation in monomer sequence (Figs. 4 and 5). Furthermore, this same sequence localizes to telomeric heterochromatin created as a result of pericentric inversions and heterochromatic additions; these karyotypic variations typify *Peromyscus* speciation, and this dynamic process of chromosome evolution would be expected to alter the relative proportions of heterochromatic repeats in each genome. Instead, we report a perpetuation of the same repeat despite extensive heterochromatic repatterning (Figs. 2, 4c, and 5).

It does not seem that the degree of intraspecific homogenization of the satellite sequence is correlated with the number of chromosome rearrangements (Figs. 2, 4a, and 5); in this study, *P. californicus* differs from the primitive *Peromyscus* karyotype by a single pericentric inversion and has an intraspecific satellite identity of 93.0%, while *P. maniculatus* differs by 15 pericentric inversions and two heterochromatic additions and *P. eremicus* differs by at least 19 heterochromatic additions and two pericentric inversions, with intraspecific homologies of 95.7% and 90.1%, respectively. Conversely, *P. aztecus* diverged from the ancestral *Peromyscus* karyotype by five pericentric inversions and yet has an intraspecific identity of only 79.2% while it shares higher interspecific homology to all species. According to the theory of molecular drive (Dover 1982), concerted evolution (intraspecific homogenization and interspecific diversity) would be expected to occur more rapidly in species with more diverged karyotypes; this is not observed in *Peromyscus*.

In addition to the conservation of this single satellite sequence throughout the *Peromyscus* genus, another unusual characteristic exists regarding *Peromyscus* chromosome evolution; while most other Rodentia lineages primarily consist of fusions, fissions, and translocations (reviewed in Romanenko et al. 2012), *Peromyscus* karyotypic evolution is characterized by a high frequency of pericentric inversions and heterochromatic additions, which do not change the chromosome number but result in varying fundamental numbers between species. These same chromosome rearrangements also typify different cytotypes within the same species. Several species show polymorphisms for one or more chromosomes; both acrocentric and biarmed chromosomes, which differ by a pericentric inversion or heterochromatic addition, are present within the same species and often as polymorphisms within a single individual (Ohno et al. 1966; Sparkes and Arakaki 1966; Te and Dawson 1971).

The number of chromosome rearrangements observed in *Peromyscus* has been shown to correlate with the extent of ecological adaptation (Arakaki et al. 1970; Bradshaw and Hsu 1972; Dixon et al. 1984; Greenbaum and Baker 1978; Loudenslager 1978; Macey and Dixon 1987; Ohno et al. 1966; Sparkes and Arakaki 1966). Pericentric inversions and heterochromatic additions result in both heterochromatic and euchromatic repatterning that would normally be deleterious to the cell, yet they are found abundantly in *Peromyscus*. Furthermore, the same karyotypic rearrangements involving heterochromatin repatterning have occurred multiple times within independent lineages of *Peromyscus*. Thus, it has been postulated that heterochromatin amplification and/or repositioning could be evolutionarily beneficial (Ohno et al. 1966; Sparkes and Arakaki 1971). Specifically, it has been previously proposed that heterochromatic repatterning (including both pericentric inversions and heterochromatic additions) may serve four hypothetical advantageous purposes by providing the following: new raw material for novel genes to emerge, spaces for safe breakpoints within euchromatic regions, the rearrangement of linkage groups, and more noncoding sequences to function in gene expression (Dixon et al. 1984). Under these models, individuals heterozygous for these repatterned chromosomes would experience an evolutionary advantage because of their genetic diversity (Sparkes and Arakaki 1971). Our data leads us to hypothesize that the homology of the satellite might allow for such beneficial rearrangements and polymorphisms to occur without gross consequence to chromosome stability. This is evidenced in the interspecific hybrid (Fig. 3), wherein we find no evidence of disruption to satellite arrays as would be predicted by studies of centromere instability in interspecific hybrids (Metcalfe et al. 2007; O’Neill et al. 1998, 2001).

A number of studies have examined the unique meiotic behavior of *Peromyscus* chromosomes, including those that are polymorphic within an individual. In autosomes, synapsis begins at the telomere of the q arm and proceeds towards the centromere (Greenbaum et al. 1986). While autosomal chromosomes that are homozygous in their heterochromatic patterning undergo usual synapsis, the heterochromatic blocks do not form chiasmata and, as such, do not partake in recombination—an observation first predicted to be true by Ohno et al. (1966). Similarly, the heterochromatic regions of autosomes polymorphic for pericentric inversions participate in “direct heterosynapsis” of the alleged nonhomologous chromatin without the formation of an inversion loop and thus do not participate in recombination. As a result, the polymorphic condition is nondeleterious in that the homologous chromosome pair may undergo meiosis without the lethal recombination of nonhomologous sequences and the subsequent production of unbalanced gametes, a finding that could account for the widespread karyotypic variation in *Peromyscus*.

From our observations, we propose a model where PMsat arrays serve as the raw material where the direct heterosynapsis between polymorphic chromosomes can occur. The satellite arrays at the centromere and pericentric region would allow for homologous synapsis to occur at these regions, regardless of the presence of pericentric inversions and/or heterochromatic additions. The same principle may be at work in hybrid or intercross Peromyscine species in which homologous chromosomes pair with each other via the alignment of the conserved satellite sequence. While the satellite itself may not be driving the speciation process, it allows for possible advantageous rearrangements to take place without mitotic or meiotic deficiencies. Thus, the karyotypic rearrangements would serve ecological and/or evolutionary purposes; as others have noted, *P. maniculatus*, which has the most rearrangements from the proposed primitive karyotype of *Peromyscus* (Fig. 2) and the highest frequency of intraspecific polymorphic chromosomes, is the most numerous, widespread, and ecologically adaptive of all species (Ohno et al. 1966; Sparkes and Arakaki 1966).

Several questions regarding satellite DNA conservation and chromosome evolution in *Peromyscus* remain elusive. First, what role—if any—the conserved satellite sequence may have in the negative selection against interchromosomal rearrangements, which are notably absent in *Peromyscus* evolution, is unknown. While the homogenous heterochromatin may be evolutionarily beneficial and allow for intrachromosomal rearrangements and polymorphic variations to exist, it is unknown what prevents the same mechanisms from causing rearrangements between nonhomologous chromosomes. Moreover, while it is possible this satellite may be conserved simply due to a lack of a diverse library of satellites present in the heterochromatin pool of the ancestor of *Peromyscus*, models of neutral evolution predict there would be a higher level of detectable change to satellite sequences simply due to drift, evidence of which is lacking in this genus. Thus, the sequence itself may serve a biological function and thus is actively maintained within the genome of each species. This observation is similar to that of a conserved satellite originally identified in cat genomes that was found to retain sequence conservation in divergent Bilateria genomes; however, unlike PMsat, the “frozen” satellite was not strictly retained at all centromeres across karyotypes nor in high-copy number tandem arrays in every species (Chaves et al. 2017). We propose that molecular drive alone does not account for the evolution of satellite DNA in *Peromyscus* and suggest that homologous arrays of satellite DNA may, either directly or indirectly, play a role in *Peromyscus* chromosome evolution.

## Abbreviations

AM: *Peromyscus aztecus hylocetes*
BP: Base pair
BW: *Peromyscus maniculatus bairdii*
CENP-A: Centromere protein A
CENP-B: Centromere protein B
DAPI: 4′,6-Diamidino-2-phenylindole dihydrochloride
dCTP: Deoxycytidine triphosphate
dUTP: Deoxyuridine triphosphate
EDTA: Ethylenediaminetetraacetic acid
EP: *Peromyscus eremicus*
FISH: Fluorescence in situ hybridization
HCl: Hydrochloric acid
HOR: Higher-order repeat
IS: *Peromyscus californicus insignis*
LL: *Peromyscus leucopus*
LP: *Peromyscus leucocephalus-subgriseus*
Na_2_HPO_4_: Sodium orthophosphate
NGS: Next-generation sequencing
PCR: Polymerase chain reaction
PMsat: *Peromyscus maniculatus* satellite repeat
PO: *Peromyscus polionotus subgriseus*
SDS: Sodium dodecyl sulfate
SM2: *Peromyscus maniculatus sonoriensis*
SSC: Saline sodium citrate
XZ: *Peromyscus melanophrys xenerus*
